# Prevalence and correlates of depressive symptoms among Chinese patients with cataracts treated in tertiary general hospitals

**DOI:** 10.7717/peerj.9397

**Published:** 2020-06-16

**Authors:** Zhong-Hua Liu, Chang-Zheng Chen, Cong Gao, De-Yi Zhou

**Affiliations:** 1 Department of Ophthalmology, Wuhan Hankou Hospital, Wuhan, China; 2 Department of Ophthalmology, Renmin Hospital of Wuhan University, Wuhan, China; 3 Department of Psychiatry, The Affiliated Wuxi Mental Health Center of Nanjing Medical University, Wuxi, China

**Keywords:** Depressive symptoms, Cataract, Epidemiology, Prevalence, Correlate, China

## Abstract

**Background:**

Previous studies recruited unrepresentative samples of Chinese patients with cataract and reported a wide range of prevalence of depressive symptoms in this patient population (18.0–89.7%). The present study determined the prevalence and correlates of depressive symptoms among a consecutive sample of Chinese patients with cataract treated in tertiary general hospitals.

**Methods:**

A total of 339 patients with cataract were consecutively selected from ophthalmology departments of two large general hospitals in Wuhan, China. Depressive symptoms were assessed with the Chinese Hospital Anxiety and Depression Scale. Logistic regression was used to identify factors that were associated with depression.

**Results:**

The prevalence of depressive symptoms was 23.9% (95% CI [19.4–28.4]%) among patients with cataract. Correlates for depressive symptoms include an education level of primary school and below (OR = 1.93, *P* = 0.038), marital status of “others” (OR =3.15, *P* < 0.001), poor family economic status (OR = 2.26, *P* = 0.010), nuclear cataract (OR =4.32, *P* < 0.001), and mixed cataract (OR = 2.76, *P* = 0.017).

**Conclusions:**

Depressive symptoms are common among Chinese patients with cataract treated in large general hospitals. Patients who are poorly educated, have a marital status other than “married”, have poor family economic status, and suffer from nuclear and mixed cataracts are at greater risk for depressive symptoms.

## Introduction

In China, cataract is the top leading cause of blindness and low vision among middle-aged and older adults, and an estimated over one-third of this population suffer from cataract (Tang et al., 2016; Tian et al., 2014; Yuan & Li, 2016). In addition to the severe functional impairments and disability due to cataract, cataract has been associated with increased risk of mental health problems such as depression and mental health services utilization (Meuleners et al., 2013). The comorbid depression further exacerbates the poor cognitive and physical health, delays recovery from surgery, and increases mortality in people with cataract (Pellegrini et al., 2020). Therefore, a greater understanding on the clinical epidemiology of depression in this patient population may help the early identification of those at risk for depression and improve their mental and physical wellbeing.

In the literature, there have been some studies investigating depression in patients with cataract (Fraser et al., 2013; Mitsonis et al., 2006; Mylona et al., 2020). For example, in Australia and Canada, researchers used Geriatric Depression Scale (GDS) to assess the severity of depressive symptoms in 329 and 672 patients awaiting cataract surgery, respectively. The two studies reported similarly high prevalence of clinically significant depressive symptoms (28.9% and 26.0%) and identified a few correlates of depression such as poor visual acuity and major medical conditions (Freeman et al., 2009; Palagyi et al., 2016). In China, to the best of our knowledge, four studies have investigated the prevalence of depression in patients with cataract (Chen et al., 2019; Chen, 2010; He & Gao, 2015; Wang et al., 2016; Zhang et al., 2018). These studies assessed depressive symptoms with a variety of self-report depression scales (i.e., GDS, Zung’s Self-rating Depression scale [SDS], 9-item Patient Health Questionnaire (PHQ-9), and Hospital Anxiety and Depression Scale (HADS)) and reported a wide range of prevalence rates: 18.0–89.7%. SDS and PHQ-9, although both are reliable for measuring depressive symptoms in the general population, they are not able to accurately evaluate depressive symptoms among patients with cataract, because they have items of somatic symptoms, which are related to aging itself and very common among patients with chronic illnesses. Due to this, there may be false-positive cases with depressive symptoms in prior studies with samples of Chinese patients with cataract. Therefore, existing studies using SDS and PHQ-9 may have overestimated the prevalence of depressive symptoms among Chinese patients with cataract. Further, these prior studies often recruited small and convenient samples and seldom considered clinical factors associated with depression in Chinese individuals with cataract.

Given the clinical importance of comorbid depression in patients with cataract and limitations in previous Chinese studies, the present study assessed the prevalence of depressive symptoms and their associated factors in a consecutive clinical sample of Chinese patients with cataract treated in tertiary general hospitals.

## Materials & Methods

### Participants

This cross-sectional study was conducted from March to July, 2019. Participants were adults receiving treatment in outpatient and inpatient departments of ophthalmology at Wuhan Hankou Hospital and Renmin Hospital of Wuhan University, two large tertiary general hospitals in Wuhan, China. By using consecutive sampling method, patients who were 18 years and older, met the diagnostic criteria for cataract after slit-lamp examination, and were capable of communicating with investigators were all invited to participate in the study. We excluded patients with eye diseases other than cataract, dementia, brain organic mental disorders, and psychotic disorders, as well as those who were too physically ill to complete the interview.

In our pilot study with a small sample of 20 patients with cataract, the prevalence of depressive symptoms was 25%. Parameters for sample size estimation of our study were: a prevalence rate of 25%, a precision of 0.05, a response rate of 0.9, and a confidence interval of 95% (95% CI). PASS 2005 (LLC, Kaysville, UT, USA) estimated that a minimum sample size of 316 would ensure the stability of our prevalence estimate.

Written informed consent was obtained from patients and their caregivers and declarations of anonymity and confidentiality had been made before the start of data collection. The Ethics Committee of Wuhan Hankou Hospital approved the study protocol (approval number: 2019-SR-Y0036).

### Procedures and instruments

Data were collected by using a standardized questionnaire. Because most patients had difficulties in reading due to visual acuity problems, the questionnaire was completed in a face-to-face and one-to-one interview manner. The study investigators were four trained master students in clinical ophthalmology. Before the formal study, 15 patients with cataract were recruited to participate in the training sessions for the investigators. The inter-rater reliability of the four investigators of the HADS depression score was satisfactory (intra-class correlation coefficient = 0.92).

Socio-demographic variables in the questionnaire were gender, age, education, marital status, current residence place (urban, rural, and urban-rural fringe), and self-rated family economic status (poor, moderate, and good).

Clinical characteristics included clinical setting (outpatient vs. inpatient), the presence of two major medical conditions (hypertension and diabetes mellitus), cataract subtype (cortical, nuclear, posterior subcapsular, and mixed), affected site (unilateral vs. bilateral), and treatment stage (before vs. after surgery). This clinical information was collected by a review of medical records.

A checklist was used to collect data on patients’ major medical conditions, which included 13 specific physical illnesses: hypertension, diabetes, heart disease, stroke and other cerebrovascular diseases, chronic obstructive pulmonary disease, cancer, tuberculosis, chronic prostatitis, chronic gastric ulcer, Parkinson’s disease, anemia, hepatic sclerosis, and arthritis.

Depressive symptoms during the past week were assessed with depression subscale of the validated Chinese HADS, which has seven items and all are answered on a 0–3 scale (Zigmond & Snaith, 1983). The total score varies between 0 and 21, with higher scores representing more severe depressive symptoms. A cut-off score of nine or higher is recommended to denote clinically significant depressive symptoms in Chinese population (Yang et al., 2014). The strength of HADS is that it can avoid reliance on somatic symptoms (i.e., pain, fatigue and insomnia) for assessing depressive symptoms (Zigmond & Snaith, 1983). Given that many patients with cataract have chronic illnesses, this tool is particularly suitable for detecting depression in cataract patients with satisfactory validity and specificity.

### Statistical analysis

Prevalence rates of depressive symptoms in the total sample and different subsamples according to socio-demographic and clinical characteristics were calculated. Comparisons of rates across subsamples were made by using Chi-square test. Multiple logistic regression model with a forward stepwise entry of significant variables in the above Chi-square test were used to identify factors associated with depressive symptoms. Odds ratios (ORs) and 95%CIs were used to quantify associations between factors and depressive symptoms. Significance level was set at 0.05 (two-sided) in all analyses. SPSS software version 12.0 was used to analyze the data.

## Results

During the study period, 370 patients were eligible for the study and all were invited to participate. Finally, a total of 339 patients with cataract completed the survey (response rate: 91.6%). Among the final sample, 47.5% were men and the average age was 67.1 years (range: 20–90, standard deviation: 10.9). Detailed socio-demographic and clinical characteristics of the study sample are displayed in Table 1.

Altogether, 81 patients were detected as having depressive symptoms. The corresponding prevalence of depressive symptoms was 23.9% (95%CI [19.4–28.4]%).

Results of comparisons across subsamples (Table 1) show that, patients having an educational attainment of primary school and below, having marital status of “others”, residing in rural area, rating their family economic status as “poor”, suffering from nuclear cataract, having bilateral cataracts, and being after surgery had significantly higher prevalence rates of depressive symptoms than their counterparts without these attributes (*P* ≤ 0.046).

In multiple logistic regression, depressive symptoms were significantly associated with an education level of primary school and below (OR =1.93, *P* = 0.038), marital status of “others” (OR = 3.15, *P* < 0.001), poor family economic status (OR = 2.26, *P* = 0.010), nuclear cataract (OR = 4.32, *P* < 0.001), and mixed cataract (OR = 2.76, *P* = 0.017) (Table 2).

## Discussion

In recent years, the importance of depression in patients with cataract and other eye diseases has been increasingly recognized, but this mental health issue remains poorly detected and managed in China’s clinical practice in ophthalmology (Li et al., 2013). One of the most important reason is that Chinese ophthalmologists are unaware of depression and its clinical characteristics among their patients. To fill this gap, we investigated the clinical epidemiology of depressive symptoms in patients with cataract. By using HADS, 23.9% of the Chinese patients with cataract were found to be depressed during the past week and the prevalence of depressive symptoms varied across different socio-demographic and clinical subgroups. Factors significantly associated with depressive symptoms in this patient population were an education level of primary school and below, marital status of “others”, poor family economic status, and two subtypes of cataract: nuclear and mixed.

**Table 1:** Socio-demographic and clinical characteristics of patients with cataract and rates of depressive symptoms according to socio-demographic and clinical variables.

Variables		Number of patients	Number of depressed patients	Rate (%)	*χ*2	*P*
Clinical setting	Outpatient	87	17	19.5		
	Inpatient	252	64	25.4	1.220	0.269
Gender	Male	161	38	23.6		
	Female	178	43	24.2	0.014	0.905
Age-group (years)	−54	36	7	19.4		
	55–64	73	12	16.4		
	65–74	148	35	23.6		
	75+	82	27	32.9	6.308	0.098
Education	Middle school and above	254	47	18.5		
	Primary school and below	85	34	40.0	16.183	<0.001
Marital status	Married	269	48	17.8		
	Others^a^	70	33	47.1	26.221	<0.001
Residence place	Urban area	246	51	20.7		
	Rural area	55	20	36.4		
	Urban-rural fringe area	38	10	26.3	6.178	0.046
Self-rated family economic status	Moderate and good^b^	253	47	18.6		
	Poor	86	34	39.5	15.503	<0.001
Hypertension	No	184	39	21.2		
	Yes	155	42	27.1	1.611	0.204
Diabetes mellitus	No	268	60	22.4		
	Yes	71	21	29.6	1.595	0.207
Cataract subtype	Cortical	121	12	9.9		
	Nuclear	113	41	36.3		
	Posterior subcapsular	31	8	25.8		
	Mixed	74	20	27.0	22.998	<0.001
Affected site	Unilateral	154	27	17.5		
	Bilateral	185	54	29.2	6.280	0.012
Treatment stage	Before surgery	117	19	16.2		
	After surgery	222	62	27.9	5.757	0.016

**Notes.**

a “Others” includes never-married, remarried, cohabitating, separated/divorced, and widowed.

b Only 9 patients rated their family economic status as “good”, so “moderate” and “good” were classified as one category.

**Table 2:** Results of multiple logistic regression on factors significantly associted with depressive symptoms in Chinese patients with cataract.

Variables		Coefficient	Standard error	Wald *χ*2	OR (95% CI)	*P*
Education	Middle school and above	1			1	
	Primary school and below	0.656	0.315	4.326	1.93 (1.04, 3.58)	0.038
Marital status	Married	1			1	
	Others^a^	1.146	0.308	13.836	3.15 (1.72, 5.75)	<0.001
Self-rated family economic status	Moderate and good^b^	1			1	
	Poor	0.814	0.317	6.618	2.26 (1.21, 4.20)	0.010
Subtype of cataract	Cortical	1			1	
	Nuclear	1.463	0.379	14.884	4.32 (2.05, 9.08)	<0.001
	Mixed	1.017	0.425	5.726	2.76 (1.20, 6.36)	0.017

**Notes.**

a “Others” includes never-married, remarried, cohabitating, separated/divorced, and widowed.

b Only 9 patients rated their family economic status as “good”, so “moderate” and “good” were classified as one category.

In China, large-scale population-based studies have shown that 5.9–8.3% of the Chinese general adults suffer from depressive symptoms (Lin et al., 2018; Tu et al., 2018; Zhou et al., 2014). Compared to these estimates in the general population, we found a much higher prevalence of depressive symptoms in Chinese patients with cataract, suggesting the high risk of depressive symptoms among patients with cataract. Compared to two previous studies that used HADS to assess the prevalence of depressive symptoms in Chinese patients with cataract (16.7–18.0% ) (Chen et al., 2019; Zhang et al., 2018), this study also demonstrated higher prevalence in our sample. This difference could be ascribed to the sample characteristics, for example, the two studies recruited patients awaiting for surgery only, but we recruited both patients before and after surgery. The significantly higher prevalence of depressive symptoms in patients after than before surgery in our study (Table 1) supports this speculation.

In line with the higher risk of depressive disorders in Chinese older adults with an educational attainment of primary school and below in comparison to those with an education level of middle school and above (Zhong et al., 2020b), we found that an educational attainment of primary school and below was significantly associated with depressive symptoms in Chinese patients with cataract. This relationship may be attributed to the poor mental health literacy of patients with a low level of education, which limits their ability to maintain and promote mental health. In general, family support from spouses can buffer the negative effect of physical illnesses such as cataract on mental health, thereby reducing the risk of depression among patients having spouses (Ivbijaro et al., 2019; Zhong et al., 2020a). This may explain the significant association between marital status of “others” and depressive symptoms in patients with cataract. Consistent with the increased risk of depression in adults of a low socio-economic status (Villarreal-Zegarra & Bernabe-Ortiz, 2020; Zhong et al., 2015), poor family financial status was significantly associated with depressive symptoms in our study. In general, persons of a low socio-economic status may have inadequate social support resources to cope with their mental health issues, resulting in elevated risk of depression among these persons.

In patients with eye diseases, researchers have found the elevated risk of depression among those with severe visual impairment (Li et al., 2013). Because patients with nuclear and mixed cataracts are more likely to have poor vision, the significant associations of depressive symptoms with nuclear and mixed cataracts are expected in our study.

This study has a few limitations. First, this is an observational study so the correlates of depressive symptoms are not, strictly speaking, risk factors. Whether or not the identified correlates cause depressive symptoms need to be examined by prospective follow-up or even interventional studies. Second, our study only measured depressive symptoms, not depressive disorders; so it is not known how many of these patients were sufficiently impaired to justify a clinical diagnosis and psychiatric treatment. Third, utilization of mental health services of patients with cataract is also essential for the development and planning of health services in clinical practice in ophthalmology, but we did not collect these data. Fourth, in general, Chinese older adults are less likely to report their depressive feelings, particularly when HADS was administered in a face-to-face interview, as what we have done in this study. This may result in an underestimation of the prevalence of depressive symptoms. Finally, some other potential factors associated with depression (smoking, exercise habit, lack of social support, length of illness, functional imperilments, failure in cataract surgery, etc.) were not assessed in the study so it remains unknown whether or not these factors would also be associated with depressive symptoms in patients with cataract.

## Conclusions

In summary, depressive symptoms are common among Chinese patients with cataract, indicating the high risk of depression in this patient population. Given the many negative impacts of depression to patients and our society, there is a pressing need to identify and address depression and other mental health problems of Chinese patients with cataract. Among patients with cataract, depressive symptoms are associated with education, marital status, economic status, and subtype of cataract. Efforts to prevent or reduce depression in clinical practice in ophthalmology may be effective to target on those who are poorly educated, are not married, have poor family economic status, and suffer from nuclear and mixed cataracts. Services for patients with cataract in clinical practice should include regular screenings for those at risk for depression and other mental health problems, expanded psychosocial supports, and, when necessary, psychiatric assessment and treatment.

##  Supplemental Information

10.7717/peerj.9397/supp-1 Supplemental Information 1 Socio-demographic and clinical variables of 339 patients with cataracts and the presence of depressive symptoms

10.7717/peerj.9397/supp-2 Supplemental Information 2 Codes of variables used in our study

## Additional Information and Declarations

### Data Availability

The following information was supplied regarding data availability: The raw data and a codebook of the variables are available in the Supplementary Files.

## References

[ref-1] Chen W, Chen G, Zheng J, Wang L (2019). Comparison of uncertainty in illness, anxiety and depression in patients with glaucoma versus patients with cataract and their related influencing factors. Acad J Chin PLA Med Sch.

[ref-2] Chen X (2010). The research of senile cataract patients’ depression situation before and after surgery. China Journal of Modern Medicine.

[ref-3] Fraser ML, Meuleners LB, Ng JQ, Morlet N (2013). Driver self-regulation and depressive symptoms in cataract patients awaiting surgery: a cross-sectional study. BMC Ophthalmology.

[ref-4] Freeman EE, Gresset J, Djafari F, Aubin MJ, Couture S, Bruen R, Laporte A, Boisjoly H (2009). Cataract-related vision loss and depression in a cohort of patients awaiting cataract surgery. Canadian Journal of Ophthalmology.

[ref-5] He J, Gao W (2015). The occurence of depression and its association with chronic diseases among community-dewlling older adults with cataract in Wuhan. Chinese Journal of Gerontology.

[ref-6] Ivbijaro G, Kolkiewicz L, Goldberg D, Riba MB, N’jie INS, Geller J, Kallivayalil R, Javed A, Švab I, Summergrad P, Laher S, Enum Y (2019). Preventing suicide, promoting resilience: is this achievable from a global perspective?. Asia-Pacific Psychiatry.

[ref-7] Li W, Zhong B, Liu X, Huang X, Dai X, Hu Q, Zhang H, Xu H (2013). Depressive symptoms among the visually disabled in Wuhan: an epidemiological survey. Shanghai Archives of Psychiatry.

[ref-8] Lin X, Yw W, Ye Y, Zhong W (2018). The status of depression and its influencing factors in adults in Fujian province. Chinese Journal of Health Statistics.

[ref-9] Meuleners LB, Hendrie D, Fraser ML, Ng JQ, Morlet N (2013). The impact of first eye cataract surgery on mental health contacts for depression and/or anxiety: a population-based study using linked data. Acta Ophthalmologica.

[ref-10] Mitsonis CI, Mitropoulos PA, Dimopoulos NP, Mitsonis MI, Andriotis NM, Gitsa OE, Mitsonis IM (2006). Anxiety and depression in cataract surgery: a pilot study in the elderly. Psychological Reports.

[ref-11] Mylona I, Floros G, Dermenoudi M, Ziakas N, Tsinopoulos I (2020). A comparative study of depressive symptomatology among cataract and age-related macular degeneration patients with impaired vision. Psychology, Health & Medicine.

[ref-12] Palagyi A, Rogers K, Meuleners L, McCluskey P, White A, Ng JQ, Morlet N, Keay L (2016). Depressive symptoms in older adults awaiting cataract surgery. Clinical and Experimental Ophthalmology.

[ref-13] Pellegrini M, Bernabei F, Schiavi C, Giannaccare G (2020). Impact of cataract surgery on depression and cognitive function: a systematic review and meta-analysis. Clinical and Experimental Ophthalmology.

[ref-14] Tang Y, Wang X, Wang J, Huang W, Gao Y, Luo Y, Yang J, Lu Y (2016). Prevalence of age-related cataract and cataract surgery in a Chinese adult population: the Taizhou eye study. Investigative Ophthalmology and Visual Science.

[ref-15] Tian F, Ren B, He Y, Jia J, Liu H, Pei J (2014). An epidemiological survey of cataract mong adults aged 50 years and above in rural, Shaanxi Province. International Eye Science.

[ref-16] Tu Q, Han A, Qin Y, Lin P, Xiang Y (2018). Prevalence of depression in adult residents and its influence factors in Jiangsu province. The Modern Medical Journal.

[ref-17] Villarreal-Zegarra D, Bernabe-Ortiz A (2020). Association between arterial hypertension and depressive symptoms: results from population-based surveys in Peru. Asia-Pacific Psychiatry.

[ref-18] Wang H, Sun HP, Wang P, Xu Y, Pan CW (2016). Cataract and depressive symptoms among older Chinese adults. Optometry and Vision Science.

[ref-19] Yang Y, Ding R, Hu D, Zhang F, Sheng L (2014). Reliability and validity of a Chinese version of the HADS for screening depression and anxiety in psycho-cardiological outpatients. Comprehensive Psychiatry.

[ref-20] Yuan Z, Li L (2016). A review on epidemiological studies of blindness and vision impariments in China. Medicine and Pharmacy of Yunnan.

[ref-21] Zhang D, Fan Z, Gao X, Huang W, Yang Q, Li Z, Lin M, Xiao H, Ge J (2018). Illness uncertainty, anxiety and depression in Chinese patients with glaucoma or cataract. Scientific Reports.

[ref-22] Zhong BL, Liu TB, Chan SS, Jin D, Hu CY, Dai J, Chiu HF (2015). Prevalence and correlates of major depressive disorder among rural-to-urban migrant workers in Shenzhen, China. Journal of Affective Disorders.

[ref-23] Zhong BL, Luo W, Xu YM, Li WX, Chen WC, Liu LF (2020a). Major depressive disorder in Chinese persons with speech disability: high rates of prevalence and perceived need for mental health care but extremely low rate of use of mental health services. Journal of Affective Disorders.

[ref-24] Zhong BL, Ruan YF, Xu YM, Chen WC, Liu LF (2020b). Prevalence and recognition of depressive disorders among Chinese older adults receiving primary care: a multi-center cross-sectional study. Journal of Affective Disorders.

[ref-25] Zhou X, Bi B, Zheng L, Li Z, Yang H, Song H, Sun Y (2014). The prevalence and risk factors for depression symptoms in a rural Chinese sample population. PLOS ONE.

[ref-26] Zigmond AS, Snaith RP (1983). The hospital anxiety and depression scale. Acta Psychiatrica Scandinavica.

